# Dynamic changes in host–virus interactions associated with colony founding and social environment in fire ant queens (*Solenopsis invicta*)

**DOI:** 10.1002/ece3.1843

**Published:** 2015-12-29

**Authors:** Fabio Manfredini, DeWayne Shoemaker, Christina M. Grozinger

**Affiliations:** ^1^ School of Biological SciencesRoyal Holloway University of LondonEghamUK; ^2^ Department of Entomology and Center for Pollinator ResearchThe Pennsylvania State UniversityUniversity ParkPennsylvania; ^3^ USDA‐ARSGainesvilleFlorida

**Keywords:** Colony founding, fire ant, gene expression, microarray, social environment, virus

## Abstract

The dynamics of host**–**parasite interactions can change dramatically over the course of a chronic infection as the internal (physiological) and external (environmental) conditions of the host change. When queens of social insects found a colony, they experience changes in both their physiological state (they develop their ovaries and begin laying eggs) and the social environment (they suddenly stop interacting with the other members of the mother colony), making this an excellent model system for examining how these factors interact with chronic infections. We investigated the dynamics of host**–**viral interactions in queens of *Solenopsis invicta* (fire ant) as they transition from mating to colony founding/brood rearing to the emergence of the first workers. We examined these dynamics in naturally infected queens in two different social environments, where queens either founded colonies as individuals or as pairs. We hypothesized that stress associated with colony founding plays an important role in the dynamics of host**–**parasite interactions. We also hypothesized that different viruses have different modalities of interaction with the host that can be quantified by physiological measures and genomic analysis of gene expression in the host. We found that the two most prevalent viruses, SINV‐1 and SINV‐2, are associated with different fitness costs that are mirrored by different patterns of gene expression in the host. In fact SINV‐2, the virus that imposes the significant reduction of a queen's reproductive output is also associated with larger changes of global gene expression in the host. These results show the complexity of interactions between *S. invicta* and two viral parasites. Our findings also show that chronic infections by viral parasites in insects are dynamic processes that may pose different challenges in the host, laying the groundwork for interesting ecological and evolutionary considerations.

## Introduction

Parasitic infections (including viruses) can reduce host fitness by increasing host mortality and by redirecting resources from the host to the parasite growth and transmission (Ebert and Bull 2008; Schmid‐Hempel 2011). The ability of the host to combat a parasitic infection may change over the course of a host's lifespan, and be compromised by the presence of other stressors. One of these stressors is reproduction in female hosts, which requires substantial resource investment in egg production and parental care. Many species have evolved physiological trade‐offs (Schmid‐Hempel 2005), where the host uses resources either to combat parasites (thereby reducing reproductive output) or to reproduce (thereby reducing antiparasite defences). These trade‐offs are particularly apparent when the host has limited access to external resources and must rely on its existing nutritional stores. When multiple, different parasites are present within the same host, the analysis of trade‐offs is more complicated because different parasites may pose unique challenges to the host (Brown et al. 2002; Mideo 2009; Natsopoulou et al. 2015).

In social insects, it is possible to examine the trade‐off between reproduction and immune response in the context of the social environment. Reproduction in many social insects (including species of ants, wasps, and bumble bees) involves independent colony founding, in which sexually mature females (queens) leave their natal colony, mate, and start a new colony, where they will lay eggs and rear brood until the first clutch of workers emerge to take over the colony tasks. Independent colony founding is a very stressful period because young queens face predation and harsh environmental conditions during mating flights, nutritional stress (in cases where queens do not forage), and the challenges associated with building a new nest and producing the first generation of brood. Furthermore, queens in many species can found colonies in pairs or groups, creating social stress as they compete over egg‐production and reproductive dominance (Bernasconi and Strassmann 1999).

Founding queens often have infections acquired from their natal colonies or from their mating partners (Schmid‐Hempel 1998). Previous studies have sought to evaluate the potential trade‐offs between reproduction and response to parasites during colony founding in bumble bees (Brown et al. 2003; Rutrecht and Brown 2008), ants (Pull et al. 2013), and termites (Calleri et al. 2007; Hartke 2010). Interestingly, studies on bumble bees indicate that during the different stages of the colony founding process, the impact of different parasites on the same host varies considerably, ranging from being highly virulent to undetectable (Rutrecht and Brown 2008), and that parasite virulence is context‐dependent and can change in response to changes in the host's conditions (Brown et al. 2003). However, the effects of social stress on antiparasitic response, interactions among different parasites, and the genomic mechanisms underlying an infection remain to be examined.

**Figure 1:**
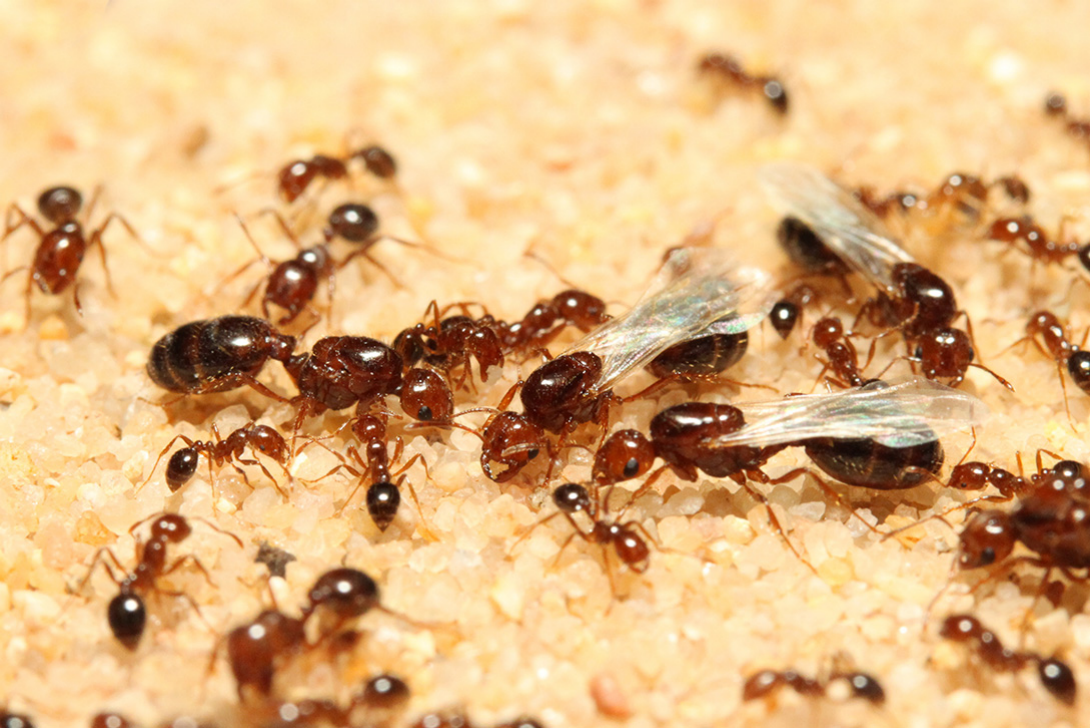
Detail of a colony of *Solenopsis invicta* Buren. The picture shows four reproductive females (larger individuals): three of them are winged and presumably unmated, while the wingless female is a newly mated queens and is ready to found a new colony. The other smaller individuals are all workers (picture by Romain Libbrecht and Yannick Wurm).

Here, we investigate the effects of colony founding and social environment on host**–**virus interactions in the invasive fire ant *Solenopsis invicta* Buren (Fig. 1). Fire ant queens of the monogyne social form (which have one functional queen per colony) found colonies independently under claustral conditions, where the queen does not forage and has no access to food until the workers emerge. Furthermore, queens may found colonies individually (haplometrosis) or in groups of two or more co‐foundresses (pleometrosis; Tschinkel 2006), where pleometrosis can be triggered by high densities of newly mated queens in a restricted area (Tschinkel and Howard 1983). Pleometrosis serves as a source of social stress because the initial cooperation among co‐foundresses quickly transitions into open conflict (Bernasconi and Keller 1996). Fire ant colonies commonly are infected by a wide range of parasites in the field, including three positive, single‐stranded picorna‐like RNA viruses named SINV‐1, SINV‐2, and SINV‐3 (Valles 2011). While fire ant host**–**virus dynamics are well‐studied in mature colonies, these dynamics and associated host trade‐offs during colony founding have not been investigated.

We used RNA virus infections of *S. invicta* as a model system to test how hosts respond to natural chronic infections that pose different challenges at different moments of fundamental biological processes such as colony founding. We hypothesized that each virus has a clear modality of interaction with the host that is quantifiable at both the physiological and genomic levels. We also hypothesized that host**–**parasite interactions are influenced by the stress associated with key steps during the colony founding process, such as ovary activation/egg‐laying and aggressive interactions between co‐foundresses in pleometrotic associations. We first examined the prevalence of SINV‐1, SINV‐2, and SINV‐3 viruses in newly mated queens (T0) and compared these estimates to infection prevalence in mature colonies collected at the same period from the same area. We then recorded viral prevalence and queen mortality at two key periods during colony founding, in both haplometrotic and pleometrotic queen associations: T1, after the first month, when queens have activated their ovaries, laid eggs and produced the first brood, and when pleometrotic associations begin to transition from cooperation to open conflict; T2, after the second month, when workers have emerged to take over colony tasks and pleometrotic associations have been reduced to monogyny by execution of all extranumerary queens (Balas and Adams 1996). We scored initial weight of newly mated queens and amount of brood produced over the first month to infer potential fitness costs associated with SINV‐1 and SINV‐2 infections – we disregarded SINV‐3 as this virus was never found associated with queens in this study. Finally, we performed a whole‐genome transcriptomic analysis of pleometrotic queens infected by SINV‐1, SINV‐2 or co‐infected with both viruses to characterize patterns of gene expression associated with viral infection and identify genes responding differentially to the two viruses. Results from our study provide a comprehensive view of the effects of stress on parasite population dynamics, infection costs and trade‐offs in their ant host, and the molecular mechanisms underpinning these host**–**parasite interactions.

## Materials and Methods

### Sample collection

Mature colonies of *Solenopsis invicta* Buren were sampled between April and May 2010 in five locations around Gainesville, Florida: Ocala (29.1845°N, 82.1409°W), Payet farm (29.7015°N, 82.4548°W), University of Florida campus (29.6437°N, 82.3555°W), near the Hilton hotel in Gainesville (29.6366°N, 82.3739°W) and Waters farm (29.3696°N, 82.2194°W). After sampling, colonies were transported to the lab and reared following standard conditions (Jouvenaz et al. 1977). For each colony we recorded the social form (monogyne or polygyne) and virus/parasite prevalence (see Molecular studies below for methods) and used these data as a comparison for data obtained from newly mated queens.

Newly mated queens were collected in a parking lot (29.6220°N, 82.3838°W) after a single nuptial mating flight occurred on May 5^th^ 2010. All 787 queens were weighed and 108 of them were immediately frozen (T0 group). The remaining 679 queens were split into two groups: 308 queens were set up in pairs (pleometrotic colony founding) based on having similar weights (range ±0.2 mg) while 371 queens were set up individually (haplometrotic colony founding). These queens were reared in the lab as previously described (Manfredini et al. 2013) to allow them to start new colonies. After worker emergence, incipient colonies were reared following standard conditions (Jouvenaz et al. 1977). Queens were sampled after 1 month (T1) and 2 months (T2). We used these sets of queens to screen for mortality during colony founding, examine levels of viruses and other parasites (see Molecular studies below) and to identify potential fitness costs associated with viral infection. Fitness costs were determined by measuring the queen's initial weight (since heavier queens typically are more likely to successfully avoid predation and establish dominance among co‐foundresses; Tschinkel 2006) and brood production at T1 (queens that produce a larger initial worker force are more likely to survive intraspecific competition; Tschinkel 2006).

Another group of 928 newly mated queens was sampled the following year (April 21^st^ 2011) in the same location as above. We weighed a subset of 446 of these queens and we arranged them in pairs based on having similar weights (range ±0.2 mg) to allow pleometrotic colony founding. These queens were sampled at T1 only because the results from the first set of assays revealed that this was the most relevant timepoint to investigate the consequences of harboring a viral infection. We estimated the numbers of viral copies and performed a transcriptomic analysis of viral infection by means of microarray analysis (see Molecular studies below) to characterize the molecular patterns associated with the presence of SINV‐1, SINV‐2 or the combined presence of both viruses.

### Statistical analyses

Statistical analyses for prevalence of viruses and fitness‐related measures were performed in R (R Development Core Team, 2007) using the packages “biology” (Murray 2008) and “MuMIn” (Barton 2014). We performed *Pearson's Chi‐squared tests* to test for associations between virus presence and modality of colony founding at T1 and T2. We used *Kruskal* **–** *Wallis chi‐squared* with *multiple Steel‐Test* and *Logistic Regression* to test for potential links between viral infection and queens' initial weight. Finally, we used *two‐factor unbalanced fixed ANOVA* to test for significant virus effect on brood production in queens at T1.

## Molecular studies

Colony social form (single queen or monogyne; multiple queens or polygyne) was determined using DNA isolated from pools of workers as described in Valles and Porter (2003) (see Data S2). DNA samples also were used to screen for the presence of two microsporidian parasites *Kneallhazia solenopsae* and *Vairimorpha invictae*; the former is found in fire ant colonies in north‐central Florida whereas the latter apparently is restricted to the native range (Valles et al. 2004, 2010). We used multiplex PCR assays to screen for viral prevalence in fire ant colonies and queens from the 2010 sample group and following Valles et al. (2009) (see Data S2). Viral copies were quantified from the *S. invicta* queens (whole bodies) collected in the 2011 sample group, using quantitative PCR (QPCR) with total RNA. We followed the protocols described in previous studies (Hashimoto et al. 2007; Hashimoto and Valles 2008) with minor modifications (see Data S2).

Microarray analyses were conducted using an aliquot of the RNA from samples above used in the QPCR experiments (2011 queen sample group). We used queens that were totally free from viral infections (*N* = 2), queens that were infected by SINV‐1 only (*N* = 3), queens infected by SINV‐2 only (*N* = 4), and queens infected by both SINV‐1 and SINV‐2 (*N* = 11). These samples were hybridized to 10 two‐color microarray chips containing more than 50K probes to unique transcripts (Roche NimbleGen, Inc., Madison WI) following a recently developed protocol (Manfredini et al. 2013, 2014). Expression data were analysed using a mixed‐model ANOVA as previously described (Manfredini et al. 2013) and using the statistical software SAS (Cary, NC): treatment, spot and dye were added as fixed effects and array was a random effect. Clustering analyses, pairwise comparisons and gene ontology analysis were performed (see Data S2) and we compared results of our study to a suite of genes identified as differentially expressed in response to Israeli Acute Paralysis Virus infection in the honey bee *Apis mellifera* (Galbraith et al. 2015).

## Results

### Prevalence of parasites

**Figure 2:**
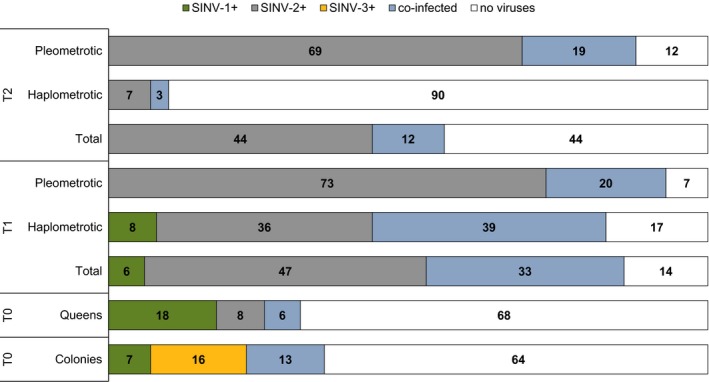
Viral prevalence detected in fire ant queens and colonies collected in northern Florida in April 2010. We surveyed workers from monogyne colonies and queens at three different timepoints: after mating flight (T0), 1 month after colony founding (T1) and 2 months after colony founding (T2). Queen data are presented both as total counts and separately for queens in haplometrotic and pleometrotic groups. The bars show proportions (% of the total) of uninfected queens (no viruses) and queens infected by SINV‐1, SINV‐2, SINV‐3, and co‐infected. NOTE: co‐infected refers to “infected by SINV‐1 and SINV‐3” in monogyne colonies, while it refers to “infected by SINV‐1 and SINV‐2” for all the other groups.

Of the 74 mature colonies that we surveyed in 2010, 45 were monogyne while 29 were polygyne (see Table S1). In monogyne colonies, SINV‐3 was the most prevalent virus (29%) followed by SINV‐1 (20%), while SINV‐2 was absent (Fig. 2). No monogyne colonies were infected with either microsporidia. A large fraction (64%) of the monogyne colonies did not harbor any of the surveyed viruses or microsporidia. In contrast, in polygyne colonies, microsporidia were highly prevalent (55% of the colonies were positive) and all three viruses were detected: SINV‐1 (55%) SINV‐2 (24%), and SINV‐3 (17%). Only 24% of polygyne colonies apparently lacked all of the surveyed viruses or microsporidia (Table S1). The trend of higher infection in polygyne colonies compared to monogyne is in agreement with a previous study where the prevalence of viral and microsporidian parasites was investigated in fire ant colonies sampled in north‐central Florida (Valles et al. 2010).

The vast majority of newly mated queens from the 2010 sample were of the *Gp‐9* ^*BB*^ genotype (106 out 108 screened or 98%), i.e. most likely produced by monogyne colonies, consistent with previous studies in this collection area (personal observation and see also Porter 1992 and Valles and Porter 2003). We screened these queens for the prevalence of parasites during three different timepoints: immediately after mating flight (T0, *N* = 108), after 1 month (T1, *N* = 51) and after 2 months (T2, *N* = 71). The two microsporidia and SINV‐3 were not detected in this study and therefore do not appear in the follow‐up analyses. SINV‐1 and SINV‐2 instead were present across the three groups of queens (Fig. 2). Infection prevalence increased dramatically from T0 (32% infected) to T1 (86% infected) and dropped again at T2 (56% infected).

The infection prevalence of SINV‐1 versus SINV‐2 varied dynamically across the timepoints. While T0 queens were more likely to be infected with SINV‐1 than SINV‐2 (18 vs. 8%) the number infected with SINV‐2 versus SINV‐1 increased dramatically in T1 (77% vs. 6%). We observed similar patterns of parasite prevalence when the infection levels were considered separately for haplometrotic and pleometrotic queens at T1 (Fig. 2) and there was no significant association between modality of colony founding and presence of viruses (χ^2^ = 1.54, df = 1, *P* = 0.2146). Finally, by T2, 43% of the queens were singly‐infected by SINV‐2, 13% were co‐infected by SINV‐1 and SINV‐2, and no queens were infected by SINV‐1 alone. Separate analyses for haplometrotic and pleometrotic queens within the T2 group revealed that the proportion of haplometrotic queens that were uninfected was significantly higher relative to the pleometrotic queens (Fig. 2) (χ^2^ = 38.8, df = 1, *P* < 0.0001).

### Queen survival and fitness costs

We evaluated survival of queens from the 2010 group during the first month. Globally, haplometrotic queens (*N* = 370) had higher survival than pleometrotic queens (*N* = 309) though the difference was not striking (73% and 69%, respectively) and the survival curves had similar patterns (Fig. S1). The slight increase of mortality among pleometrotic queens starting from day 21 reflects the beginning of lethal fights between co‐foundresses at that timepoint.

**Figure 3:**
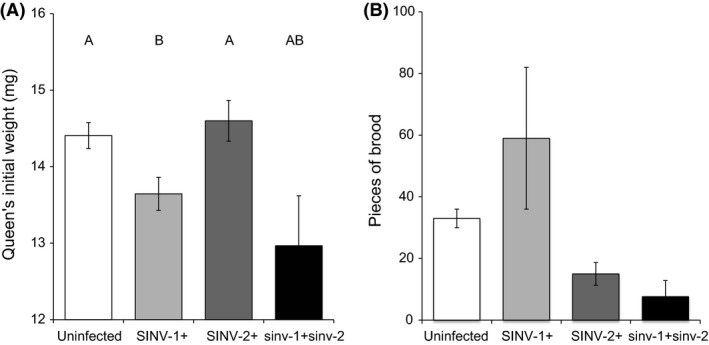
Fitness costs associated with viral infection in fire ant queens. (A) Average weight (±SE) of uninfected queens (*N* = 73) and queens infected by SINV‐1 (*N* = 20), SINV‐2 (*N* = 9) and co‐infected by both viruses (*N* = 6) as measured immediately after mating flight. Different letters above bars indicate statistical significance (Kruskal–Wallis chi‐squared with multiple Steel‐Test, *P* < 0.05). (B) Average numbers of brood (±SE) produced by uninfected queens (*N* = 2) and queens infected by SINV‐1 (*N* = 2), SINV‐2 (*N* = 4) and co‐infected (*N* = 3) as detected after 1 month from colony founding. Only infection by SINV‐2 had a significant effect on brood production (two‐factor unbalanced fixed ANOVA,* P* < 0.01).

We also evaluated initial weight of these queens at T0 as a first measure associated with fitness costs due to viral infection (Fig. 3). Queens harboring SINV‐1 (*N* = 20) weighed significantly less than uninfected queens (*N* = 73) or queens infected by SINV‐2 (*N* = 9), while coinfected queens (*N* = 6) were intermediate (Kruskal**–**Wallis chi‐squared with multiple Steel‐Test = 11.0297, df = 3, *P* = 0.01157; significantly different: SINV‐1 vs. control *P* = 0.028 and SINV‐1 vs. SINV‐2 *P* = 0.042; other comparisons *P* > 0.05). Logistic regression analysis also confirmed the significant association between SINV‐1 infection and lower weight (*b* = −0.508, *z* = −2.848, *P* = 0.004).

Finally, we investigated whether harboring a viral infection had a measurable effect on brood production (number of eggs, larvae, and pupae) in a subset of 11 haplometrotic queens at T1 after counting the total number of pieces of brood in the colony under a dissection microscope (Fig. 3). We observed a significant effect only for SINV‐2. Queens infected by this virus produced significantly fewer pieces of brood compared with queens lacking SINV‐2 (*F* = 15.195, df = 1, *P* = 0.006). There was no significant effect of SINV‐1 infection or co‐infection on brood production (respectively, *F* = 1.1014, df = 1, *P* = 0.329 and *F* = 3.512, df = 1, *P* = 0.103).

### Quantification of viral loads

We screened 44 pleometrotic queens from the 2011 sample with QPCR to quantify the number of genome copies for SINV‐1 and SINV‐2 in whole bodies at T1. The majority of these queens (34, i.e. 77%) were infected by both viruses while only three queens were infected by SINV‐1 alone (7%), four were infected by SINV‐2 alone (9%) and three were not infected at all (7%). Interestingly, SINV‐1 was always present with much higher copy numbers than SINV‐2, in either singly infected or coinfected queens (Fig. S2). Average genome copies in infected queens were 224,266 for SINV‐1 and 74 for SINV‐2.

### Transcriptomic analysis of viral infection

**Figure 4:**
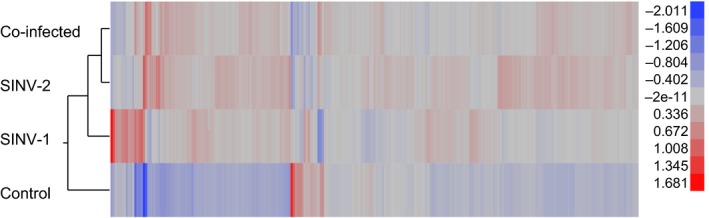
Hierarchical clustering of differentially expressed transcripts associated with viral infections in fire ant queens. Clustering was performed on the 247 transcripts significantly differentially expressed (FDR < 0.05) in at least one of the pairwise comparisons across treatment groups. Red‐colored transcripts are up‐regulated while blue‐colored are down‐regulated.

We performed microarray analysis on pleometrotic queens collected at T1 in the 2011 sample group, comparing uninfected queens with queens infected with SINV‐1, SINV‐2, and both viruses. Overall, 247 unique transcripts were significantly differentially expressed (FDR < 0.05) between at least two treatment groups (Table S2). We used this set of transcripts to perform global analysis of gene expression across treatment groups. Hierarchical clustering showed that all infected queens (SINV‐1, SINV‐2, and co‐infected) clustered together and formed a separate macro‐group compared to controls lacking these viruses (Fig. 4). SINV‐2 infected and co‐infected queens further clustered to form a smaller subgroup suggesting that the presence of SINV‐2 is the major factor driving gene expression differences in co‐infected queens, despite the much lower viral titers of this virus compared to SINV‐1. Most of the significantly different transcripts were up‐regulated in infected queens relative to controls, as shown by a K‐means clustering analysis (Fig. S3). Gene ontology analysis of the 247 unique differentially expressed transcripts identified 39 annotation clusters (Table S3) and four significantly enriched GO terms (*P* < 0.05, Table S4), including the molecular function ‘exonuclease activity’ and the following biological processes: primary metabolic process, RNA binding, and nuclear‐transcribed mRNA catabolic process.

**Figure 5:**
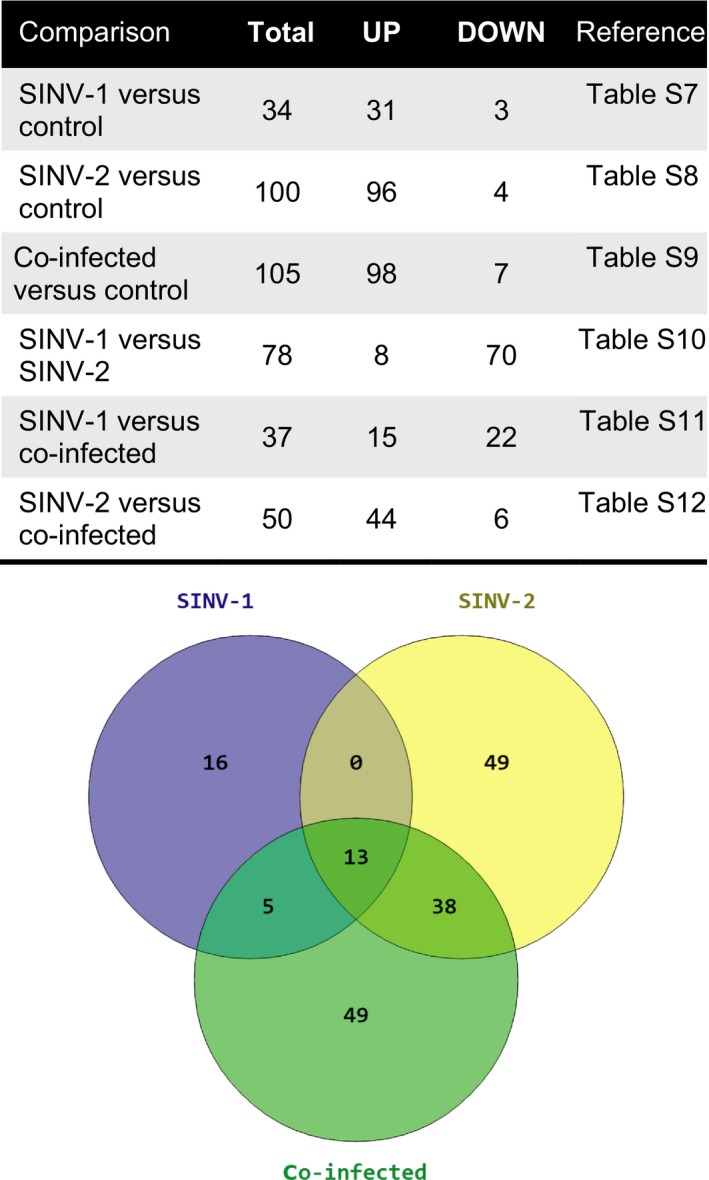
Pairwise comparisons of transcripts differentially expressed across groups. TOP: number of transcripts that were significantly differentially expressed (total) and up‐ or down‐regulated in each of the six pairwise comparisons across treatment groups. BOTTOM: overlap analysis of transcripts that were significantly differentially expressed in each group of infected queens as compared to controls.

We performed pairwise comparisons between treatment groups (Fig. 5 and Tables S7–S12). We found 13 transcripts that were differentially regulated in infected queens overall compared to controls (Table S6), including the *Drosophila* orthologs that may function in mediating viral entry into cells, viral replication, and cell function: *Negative elongation factor D* (NELF), which is an inhibitor of transcription possibly via pausing of RNA Polymerase II (Missra and Gilmour 2010), *α‐Mannosidase class I b* (*α‐Man‐Ib*) which functions in the *Drosophila* encapsulation response to wasp parasitoids (Mortimer et al. 2012), *kekkon5* (*kek5*), which is a transmembrane protein containing leucine‐rich repeats (LRRs) and controls the Bone Morphogenetic Protein (BMP) signaling pathway (Evans et al. 2009), *Transport and Golgi organization 9* (*Tango9*), which is involved in protein secretion and Golgi organization (Bard et al. 2006) and *Dmel_CG8128*, which is a regulator of cell cycle (Guest et al. 2011).

Finally, we compared the same 247 unique transcripts (corresponding to 167 *Drosophila* orthologs) to the list of 753 genes (corresponding to 307 unique *Drosophila* orthologs) that were significantly differentially expressed in honey bees infected by Israeli Acute Paralysis Virus (Galbraith et al. 2015). There were 11 genes in common between the two studies (Table S5): this overlap was larger than expected of two independent groups but not statistically significant (hypergeometric test: representation factor: 1.3, *P *=* *0.249). Several of these genes have important functions in *Drosophila*:* Phosphogluconate dehydrogenase* and *cytohesin‐1‐like* are involved in the activation of the mitogen‐activated protein kinase (MAPK) (Hahn et al. 2013), *Juvenile hormone epoxide hydrolase 2* (*Jheh2*) mediates the catabolism of juvenile hormone (Share and Roe 1988) (but has been co‐opted in *Apis mellifera* to regulate dietary lipid catabolism; Mackert et al. 2010), *toll like receptor 1* (*Toll*‐*1*) is an important player in the antimicrobial humoral response and regulation of haemocyte differentiation (Lemaitre et al. 1996; Zettervall et al. 2004), *Death resistor Adh domain containing target* (*Drat*) is involved in apoptosis and response to hypoxia (Azad et al. 2009; Chen et al. 2012), *disc large 1* (*dlg1*) regulates complex behaviors including phototaxis and circadian activity (Mendoza‐Topaz et al. 2008), *clockwork orange* (*cwo*) and *Dmel_CG5237* (*unc79*) also operate in circadian clock neurons to promote rhythmic behavior (Richier et al. 2008; Lear et al. 2013), while *Formin‐like protein* (*Dmel_CG32138*) and *Dmel_CG17912* are involved in neurogenesis (Sepp et al. 2008; Iyer et al. 2013).

## Discussion

We examined how host**–**parasite interactions (including trade‐offs between immune function and reproduction) are modulated during stressful periods of a fire ant queen's life cycle, and under different social conditions. We found that two viruses, SINV‐1 and SINV‐2, were common across the 2‐month study period, though their prevalence changed considerably over time. Single haplometrotic queens mostly cleared their viral infections by the end of the founding process whereas a high proportion of queens in paired pleometrotic groups remained infected. The two viruses appear to affect their hosts differently: SINV‐1 infected queens weigh less than uninfected queens whereas SINV‐2 infected queens do not, but the latter produce far less brood. SINV‐2 is the major driver of gene expression though SINV‐1 is present at much larger copy numbers. Below we discuss these findings and implications to fire ant biology and host**–**parasite interactions.

### Infection prevalence and patterns in queens and colonies over time

Variation between the proportions of viruses in newly mated queens and colonies suggest that the routes of transmission of the three SINV viruses may be complex. The queens primarily were derived from monogyne colonies. SINV‐3 was the most prevalent virus (29%) in the sampled monogyne colonies, followed by SINV‐1 (20%), while SINV‐2 was absent. However, SINV‐3 was absent in newly mated queens (collected few hours after they left their natal colony), and SINV‐1 and SINV‐2 were both present at low levels (18% vs. 8%). These differences may reflect differences in queen versus worker susceptibility to these viruses (since workers were sampled to assess colony infection levels). Indeed, previous work has shown that workers have higher viral loads of SINV‐1 and SINV‐2 than queens sampled in the same colony (Valles 2011). For SINV‐3 instead, similar levels of infections have been detected in queens and workers (Valles and Hashimoto 2009). Multiple factors could explain the complete absence of this virus in the queens that we screened, including the possibility that infected queens are unable to navigate effectively and reach the same landing sites as SINV‐3‐free queens.

The proportion of queens infected by SINV‐2 sharply increased from T0 to T1. An external source for new viral infections is unlikely because queens in this time were confined within claustral nests with no access to water or food resources. Both haplometrotic and pleometrotic queens showed similar increases in SINV‐2 levels, suggesting that it was not simply a result of horizontal transfer between the pleometrotic queens. It is more likely that SINV‐2 actually infects a large proportion of queens at T0, but the infection levels were below the detection threshold, and rose significantly by T1. Since queens were actively laying eggs between T0 and T1, these results could indicate a possible trade‐off between reproduction and immune function, resulting in increased SINV‐2 titres. Such trade‐offs have been observed repeatedly in invertebrates (Schmid‐Hempel 2003).

There was a general reduction in the proportion of infected queens at T2, and this reduction was greater in the haplometrotic group. The first generation of workers emerges between T1 and T2, and these workers will initiate foraging and supply the queen with food. Thus, by T2, the presumed trade‐off between immunity and reproduction is likely mitigated because nutritional resources are no longer limited, allowing the queens to increase their immune function and combat viral infections. Interestingly, there were significantly more pleometrotic queens infected with viruses than haplometrotic queens at T2, suggesting that the stress of reproductive competition in the former group undermines the queen's ability to mount an effective immune response (but note that at T2 all pleometrotic couples had been reduced to monogyny by execution of one queen). This observation seems to parallel data on infection rates in polygyne colonies (Valles et al. 2010), an analogous scenario where multiple queens coexist within the same nest. However, it is not possible to directly link colony founding by pleometrosis to polygyny, because pleometrotic queens generally derive from monogyne colonies and experience a social environment that is significantly more stressful.

### Different infection modalities for SINV‐1 and SINV‐2

Previous studies have suggested that SINV‐1 and SINV‐2 viruses cause similar chronic and mostly asymptomatic infections in fire ants, which may result in mortality under certain stressful conditions (Valles 2011). This appears to be a common pattern observed for other arthropod‐infecting positive, single‐stranded RNA viruses, as for example in the honey bee (Chen and Siede 2007). However, our results demonstrate that the two viruses interact differently with their fire ant host and are associated with measurable (and distinct) fitness costs and significant gene expression changes within their host. These features are more typical of an acute infection and may be caused by the stressful conditions of the colony founding process or the laboratory rearing conditions.

SINV‐1 appears to play a role at an early stage of a queen's life cycle, from development in her natal colony up to or shortly after her nuptial mating flight. SINV‐1 infected queens weighed significantly less at T0, and this likely would reduce the probability of successful colony founding. Earlier studies have shown that queen weight is positively correlated with reproductive potential, with resistance to stress and also with fighting ability against rival nestmates or other ants competing for nest sites in the same area (Hood and Tschinkel 1990; Tschinkel 2006). An open question for future study is whether reduced queen weight is due to the infection itself (i.e., the virus prevents the queen from properly storing nutrients before mating flight), whether lighter queens are the product of colonies with chronic infection where the network of food distribution is less efficient in general or whether queens that weigh less (and therefore have fewer energy reserves) are more likely to become infected.

SINV‐2 instead appears to play a role at a later stage of colony founding, at T1, resulting in infected queens producing less brood. Brood production is another fundamental component of successful colony founding, as the early emergence of a functional worker force provides the nest with the food and defense necessary to survive in the field (Porter and Tschinkel 1986). Other interesting observations on fitness costs of viral infections confirm that SINV‐2 has a detrimental effect on fire ant incipient colonies: colonies harboring this virus have longer claustral periods, weigh less and show slower growth (Mark Fisher unpublished data). All of these features typically result in a less competitive colony in the field. It will be of interest in the future to test whether newly mated queens infected by SINV‐2 preferentially join pleometrotic associations instead of founding a colony alone. Such behavior potentially would increase chances that queens successfully establish new colonies thanks to contributions of partner queens (provided that these are instead free of SINV‐2) that may buffer the detrimental effects associated with infection.

The gene expression data confirmed that SINV‐2 infection is the major driver of gene expression patterns at the T1 timepoint of a queen's life cycle. SINV‐2 was associated with a larger change in the gene expression patterns compared with SINV‐1, despite being present at lower genome copy numbers (~three orders of magnitude as suggested by QPCR). However, recent studies demonstrated that 1–20 copies of viral genomes are sufficient to establish cell infection in several host**–**parasite systems (Miyashita et al. 2015). Most of the genes that were differentially expressed between SINV‐1 and SINV‐2 infected queens are up‐regulated in SINV‐2 infected queens (see Data S1). Two of these genes *ATPsyn‐b* and *α‐Man‐Iib* are immune effectors, suggesting that ant queens may respond in different ways to SINV‐1 and SINV‐2 infection (i.e. by mounting a stronger immune response toward SINV‐2 while tolerating SINV‐1 better). Other genes that were differentially expressed in the presence of the two viruses are genes involved in methylation (*ash2*,* csul*), autophagy (*Tango7, PEK*), and regulation of transcription (*Doc1, NC2β*). These biological functions have been linked to the antiviral response of honeybees to Israeli Acute Paralysis Virus in a recent study (Galbraith et al. 2015). Additionally, genes associated with diet and fat body metabolism (*CdsA*,* Hr96*,* Cul1*,* bgm*,* schlank*) and reproductive activation (*CkIIα*,* nos*,* retn*,* fz2*) were differentially regulated in SINV‐1 and SINV‐2 infected queens. These expression differences could be associated with the differential brood production we recorded in SINV‐1 versus SINV‐2 infected queens. Finally, genes linked to neurogenesis and sensory perception showed different patterns of expression in queens infected by the two viruses (*cta*,* nonA*,* RhoGAPp190*,* CG11180*,* CG6807*). These genes potentially are linked to different behavioral responses to viral infection in SINV‐1 and SINV‐2 infected queens. Unfortunately, these types of data are still missing for fire ant queens, but there is some preliminary evidence that the third fire ant virus, SINV‐3, impacts workers' behavior by preventing them from acquiring and/or distributing solid food to the larvae (Valles et al. 2014).

Interestingly, many of the genes significantly differentially expressed across treatment groups are involved in primary metabolism and transcription activity. Most of these genes are up‐regulated in infected queens, which suggests that viral infections cause an increase in both the metabolic and transcription activities within the host. Such processes could be triggered either by the viral particles replicating within the cells of the host or by the host itself assembling and releasing antiviral effectors in an attempt to clear the infection. Analogously, *Drosophila* viruses such as Flock House, Nora, Sindbis and Vesicular Stomatitis trigger the up‐regulation of host genes (Castorena et al. 2010; Xu et al. 2012; Cordes et al. 2013), and similar results were found in honey bees infected with Israeli Acute Paralysis Virus (Galbraith et al. 2015). However, genes up‐regulated by these different viruses are not the same. Indeed, very few genes were shared between our study and studies of transcriptional responses to virus infection in *A. mellifera* (see Results and Table S5) and *D. melanogaster* (data not shown). These differences potentially could result from variation in the co‐evolutionary history between host and parasite, different modalities of parasite infection (e.g. acute vs. chronic or natural vs. lab infection) or methodological differences in the experimental designs (e.g. microarrays vs. RNAseq).

## Conclusions

Our results reveal the complexity of host**–**parasite interactions in cases where multiple parasites coexist within the same host and live in association with them for a long period and across important life‐history stages. The dynamics of viral infections in fire ants appear to be quite plastic and respond to multiple internal and external factors in the host, including its behavior, physiological state, and social environment. Stress associated with colony founding and trade‐offs among competition, immunity and reproduction clearly play key roles in regulating infection dynamics. Finally, the transcriptional responses of the host correlate with the observed fitness consequences such that the virus with the greater fitness consequences at that time also elicits the highest transcriptional response, despite having lower overall copy numbers. It will be of great interest in the future to disentangle how viral infections and the social environment interact at the level of the host's transcriptome, by comparing the response to the infection in pleometrotic and haplometrotic queens. This study lays the necessary groundwork for studying dynamic changes in host**–**parasite interactions across social insect life stages and social conditions in an ecological and evolutionary perspective.

## Data accessibility

The array data were deposited on the ArrayExpress website according to MIAME standards and are available in the ArrayExpress database (www.ebi.ac.uk/arrayexpress) under accession number E‐MTAB‐3581. Gene expression data are provided as supplementary tables with this article.

## Conflict of Interest

None declared.

## Supporting information


**Data S1.** Genes associated with SINV‐1 and SINV‐2 infections.

**Data S2.** Detailed methods for molecular studies.

**Figure S1**. Survival curves for founding queens during the first month of activity.

**Figure S2.** Copy numbers of SINV‐1 and SINV‐2 in queens that harbour one of the viruses or both of them as detected by quantitative real‐time PCR.

**Figure S3.** K‐means clustering analysis of significantly differentially expressed transcripts.

**Table S1.** Screening of field colonies for parasite prevalence and social form.

**Table S2.** Raw output of SAS statistical analysis of gene expression for microarray data.

**Table S3.** Gene ontology analysis of significantly differentially expressed transcripts by means of functional annotation clustering in DAVID.

**Table S4.** Gene lists that were enclosed in the four significant clusters produced by analysis in DAVID.

**Table S5.** Results of comparative studies with genes that were differentially expressed in honey bees infected by Israeli Acute Paralysis Virus (Galbraith et al. 2015 study).

**Table S6.** Transcripts that were differentially expressed in SINV‐1, SINV‐2 and co‐infected queens vs. controls.

**Table S7.** Significantly differentially expressed transcripts between SINV‐1 infected queens and controls.

**Table S8.** Significantly differentially expressed transcripts between SINV‐2 infected queens and controls.

**Table S9.** Significantly differentially expressed transcripts between co‐infected queens and controls.

**Table S10.** Significantly differentially expressed transcripts between SINV‐1 and SINV‐2 infected queens.

**Table S11.** Significantly differentially expressed transcripts between SINV‐1 and co‐infected queens.

**Table S12.** Significantly differentially expressed transcripts between SINV‐2 and co‐infected queens.
