# Supplementary material for: Dynamic changes in host–virus interactions associated with colony founding and social environment in fire ant queens (Solenopsis invicta)
Source: Ecol Evol. 2015 Dec 29;6(1):233–44. doi: 10.1002/ece3.1843 (PMC4716520; doi:10.1002/ece3.1843)
Supplement: Supplementary file 1 — Data S1. Genes associated with SINV‐1 and SINV‐2 infections. [file ECE3-6-233-s001.pdf]

Genes associated with SINV-1 and SINV-2 infections

	Drosophila ortholog	*Gene symbol*	Biological function	Ref	*S. invicta* gene	Estimate
IMMUNITY	*ATP synthase, subunit B*	*ATPsyn-b*	phagocytosis	(Stroschein-Stevenson et al., 2006)	SINVlc3_039069	-0.5806
	α-Mannosidase class II b	*α-Man-Iib*	encapsulation response	(Mortimer et al., 2012)	SINVlc3_015687	-1.3932
METHYLATION	*capsuleen*	*csul*	methyltransferase activity	(Gonsalvez et al., 2006)	SINVlc3_015973	-0.9694
	absent, small, or homeotic discs 2	*ash2*	histone H3-K4 methylation	(Mohan et al., 2011)	SI2.2.0_13294	-0.6947
AUTOPHAGY	*pancreatic eIF-2alpha kinase*	*PEK*	positive regulation of autophagy	(Nagy et al., 2013)	SI2.2.0_04925	1.2945
	Transport and Golgi organization 7	*Tango7*	regulation of apoptotic signalling pathway	(D'Brot et al., 2013)	SINVlc3_025162	-0.9321
TRANSCRIPTION	*Dorsocross1*	*Doc1*	regulation of gene expression	(Stöbe et al., 2009)	SINVlc3_010276	-1.1348
	Protein Dr1; Negative Cofactor 2β	*NC2β*	transcription factor	(Willy et al., 2000)	SINVlc3_030042	-1.003
FAT BODIES	*CDP diglyceride synthetase*	*CdsA*	lipid storage	(Liu et al., 2014)	SINVlc3_026357	1.526
	Hormone receptor-like in 96	*Hr96*	triacylglycerol and cholesterol metabolism	(Sieber and Thummel, 2012)	SINVlc3_024099	-0.7612
	Cullin 1	*Cul1*	InR/PI3K/TOR pathways	(Wong et al., 2013)	SI2.2.0_13032	-0.5181
	bubblegum	*bgm*	long-chain fatty acid metabolic process	(Min and Benzer, 1999)	SINVlc3_031713	-0.6886
	Longevity assurance gene 1	*schlank*	body fat metabolism	(Bauer et al., 2009)	SI2.2.0_13166	-0.982
REPRODUCTION	*casein kinase IIα*	*CkIIα*	response to starvation; circadian rhythms; oogenesis	(Ding et al., 2014, Lin et al., 2005, Wong et al., 2011)	SINVlc3_042716	-0.9853
	nanos	*nos*	oogenesis	(Becalska et al., 2011)	SINVlc3_017341	-0.9608
	retain	*retn*	oogenesis; regulation of female receptivity	(Schupbach and Wieschaus, 1991, Ditch et al., 2005)	SINVlc3_016720	-0.6532
	frizzled 2	*fz2*	ovarian development	(Cohen et al., 2002)	SINVlc3_024002	-0.7077
NEURO-SENSORIAL	*concertina*	*cta*	D5 dopamine receptor binding	N/A	SINVlc3_032097	-0.8345
	no on or off transient A	*nonA*	visual perception	(Rendahl et al., 1992)	SI2.2.0_09391	-1.7529
	Rho GTPase activating protein p190	*RhoGAPp190*	mushroom body development	(Billuart et al., 2001)	SINVlc3_029401	-0.5636
	Dmel_CG11180	*CG11180*	neurogenesis	(Neumüller et al., 2011)	SI2.2.0_08138	-0.6541
	Formin-like protein CG32138	*CG6807*	neuron projection morphogenesis	(Sepp et al., 2008)	SINVlc3_032090	-0.6422
OTHERS	*Death resistor Adh domain containing target*	*Drat*	cellular response to ethanol; response to hypoxia	(Chen et al., 2012)	SINVlc3_012825	-0.4861
	Dmel_CG5237	*unc79*	locomotory rhythm	(Lear et al., 2013)	SINVlc3_007105	-0.4946
	PICK1 ortholog	*PICK1*	neuroendocrine system; regulated secretory pathway	(Jansen et al., 2009)	SINVlc3_038018	-0.7021
	Poly(A) RNA polymerase gld-2 homolog A	*Gld2*	long-term memory	(Kwak et al., 2008)	SINVlc3_018308	-0.7352

Note: positive “Estimate” means up-regulated in SINV-1 infected, while negative “Estimate” means up-regulated in SINV-2 infected.

References

BAUER, R., VOELZMANN, A., BREIDEN, B., SCHEPERS, U., FARWANAH, H., HAHN, I., ECKARDT, F., SANDHOFF, K. & HOCH, M. 2009. Schlank, a member of the ceramide synthase family controls growth and body fat in *Drosophila*. *The EMBO Journal,* 28**,** 3706-3716.

BECALSKA, A. N., KIM, Y. R., BELLETIER, N. G., LERIT, D. A., SINSIMER, K. S. & GAVIS, E. R. 2011. Aubergine is a component of a nanos mRNA localization complex. *Developmental biology,* 349**,** 46-52.

BILLUART, P., WINTER, C. G., MARESH, A., ZHAO, X. & LUO, L. 2001. Regulating axon branch stability. The role of p190 RhoGAP in repressing a retraction signaling pathway. *Cell,* 107**,** 195-207.

CHEN, P., TU, X., AKDEMIR, F., CHEW, S. K., ROTHENFLUH, A. & ABRAMS, J. M. 2012. Effectors of alcohol-induced cell killing in *Drosophila*. *Cell Death and Differentiation,* 19**,** 1655-1663.

COHEN, E. D., MARIOL, M. C., WALLACE, R. M. H., WEYERS, J., KAMBEROV, Y. G., PRADEL, J. & WILDER, E. L. 2002. DWnt4 regulates cell movement and focal adhesion kinase during *Drosophila* ovarian morphogenesis. *Developmental Cell,* 2**,** 437-448.

D'BROT, A., CHEN, P., VAISHNAV, M., YUAN, S., AKEY, C. W. & ABRAMS, J. M. 2013. Tango7 directs cellular remodeling by the *Drosophila* apoptosome. *Genes & Development,* 27**,** 1650-1655.

DING, F., GIL, M. P., FRANKLIN, M., FERREIRA, J., TATAR, M., HELFAND, S. L. & NERETTI, N. 2014. Transcriptional response to dietary restriction in *Drosophila melanogaster*. *Journal of Insect Physiology,* 69**,** 101-106.

DITCH, L. M., SHIRANGI, T., PITMAN, J. L., LATHAM, K. L., FINLEY, K. D., EDEEN, P. T., TAYLOR, B. J. & MCKEOWN, M. 2005. *Drosophila* retained/dead ringer is necessary for neuronal pathfinding, female receptivity and repression of fruitless independent male courtship behaviors. *Development,* 132**,** 155-164.

GONSALVEZ, G. B., RAJENDRA, T. K., TIAN, L. & MATERA, A. G. 2006. The Sm-protein methyltransferase, dart5, is essential for germ-cell specification and maintenance. *Current Biology,* 16**,** 1077-1089.

JANSEN, A. M., NÄSSEL, D. R., MADSEN, K. L., JUNG, A. G., GETHER, U. & KJAERULFF, O. 2009. PICK1 expression in the *Drosophila* central nervous system primarily occurs in the neuroendocrine system. *Journal of Comparative Neurology,* 517**,** 313-332.

KWAK, J. E., DRIER, E., BARBEE, S. A., RAMASWAMI, M., YIN, J. C. & WICKENS, M. 2008. GLD2 poly (A) polymerase is required for long-term memory. *Proceedings of the National Academy of Sciences,* 105**,** 14644-14649.

LEAR, B. C., DARRAH, E. J., ALDRICH, B. T., GEBRE, S., SCOTT, R. L., NASH, H. A. & ALLADA, R. 2013. UNC79 and UNC80, putative auxiliary subunits of the narrow abdomen ion channel, are indispensable for robust circadian locomotor rhythms in *Drosophila*. *PLoS ONE,* 8**,** e78147.

LIN, J. M., SCHROEDER, A. & ALLADA, R. 2005. In vivo circadian function of casein kinase 2 phosphorylation sites in *Drosophila* PERIOD. *Journal of Neuroscience,* 25**,** 11175-11183.

LIU, Y., WANG, W., SHUI, G. & HUANG, X. 2014. CDP-Diacylglycerol Synthetase Coordinates Cell Growth and Fat Storage through Phosphatidylinositol Metabolism and the Insulin Pathway. *PLoS Genetics,* 10**,** e1004172.

MIN, K. T. & BENZER, S. 1999. Preventing neurodegeneration in the *Drosophila* mutant bubblegum. *Science,* 284**,** 1985-1988.

MOHAN, M., HERZ, H. M., SMITH, E. R., ZHANG, Y., JACKSON, J., WASHBURN, M. P., FLORENS, L., EISSENBERG, J. C. & SHILATIFARD, A. 2011. The COMPASS Family of H3K4 Methylases in *Drosophila*. *Molecular and Cellular Biology,* 31**,** 4310-4318.

MORTIMER, N. T., KACSOH, B. Z., KEEBAUGH, E. S. & SCHLENKE, T. A. 2012. Mgat1-dependent N-glycosylation of Membrane Components Primes *Drosophila melanogaster* Blood Cells for the Cellular Encapsulation Response. *PLoS Pathogens,* 8**,** e1002819.

NAGY, P., VARGA, A., PIRCS, K., HEGED?S, K. & JUHÁSZ, G. 2013. Myc-Driven Overgrowth Requires Unfolded Protein Response-Mediated Induction of Autophagy and Antioxidant Responses in *Drosophila melanogaster*. *PLoS Genetics,* 9**,** e1003664.

NEUMÜLLER, R. A., RICHTER, C., FISCHER, A., NOVATCHKOVA, M., NEUMÜLLER, K. G. & KNOBLICH, J. A. 2011. Genome-Wide Analysis of Self-Renewal in *Drosophila* Neural Stem Cells by Transgenic RNAi. *Cell stem cell,* 8**,** 580-593.

RENDAHL, K., JONES, K., KULKARNI, S., BAGULLY, S. & HALL, J. 1992. The dissonance mutation at the no-on-transient-A locus of *D. melanogaster:* genetic control of courtship song and visual behaviors by a protein with putative RNA-binding motifs. *The Journal of neuroscience,* 12**,** 390-407.

SCHUPBACH, T. & WIESCHAUS, E. 1991. Female sterile mutations on the second chromosome of *Drosophila melanogaster*. *Genetics,* 129**,** 1119-1136.

SEPP, K. J., HONG, P., LIZARRAGA, S. B., LIU, J. S., MEJIA, L. A., WALSH, C. A. & PERRIMON, N. 2008. Identification of neural outgrowth genes using genome-wide RNAi. *PLoS Genetics,* 4**,** e1000111.

SIEBER, M. H. & THUMMEL, C. S. 2012. Coordination of Triacylglycerol and Cholesterol Homeostasis by DHR96 and the *Drosophila* LipA Homolog magro. *Cell Metabolism,* 15**,** 122-127.

STÖBE, P., STEIN, M. A., STEIN, S. M., HABRING-MÜLLER, A., BEZDAN, D., FUCHS, A. L., HUEBER, S. D., WU, H. & LOHMANN, I. 2009. Multifactorial regulation of a hox target gene. *PLoS Genetics,* 5**,** e1000412.

STROSCHEIN-STEVENSON, S. L., FOLEY, E., O'FARRELL, P. H. & JOHNSON, A. D. 2006. Identification of *Drosophila* gene products required for phagocytosis of *Candida albicans*. *PLoS Biology,* 4**,** e4.

WILLY, P. J., KOBAYASHI, R. & KADONAGA, J. T. 2000. A basal transcription factor that activates or represses transcription. *Science,* 290**,** 982-985.

WONG, J. J., LI, S., LIM, E. K., WANG, Y., WANG, C., ZHANG, H., KIRILLY, D., WU, C., LIOU, Y. C., WANG, H. & YU, F. 2013. A Cullin1-Based SCF E3 Ubiquitin Ligase Targets the InR/PI3K/TOR Pathway to Regulate Neuronal Pruning. *PLoS Biology,* 11**,** e1001657.

WONG, L. C., COSTA, A., MCLEOD, I., SARKESHIK, A., YATES, J., KYIN, S., PERLMAN, D. & SCHEDL, P. 2011. The Functioning of the *Drosophila* CPEB Protein Orb Is Regulated by Phosphorylation and Requires Casein Kinase 2 Activity. *PLoS ONE,* 6**,** e24355.
