# Supplementary material for: Dynamic changes in host–virus interactions associated with colony founding and social environment in fire ant queens (Solenopsis invicta)
Source: Ecol Evol. 2015 Dec 29;6(1):233–44. doi: 10.1002/ece3.1843 (PMC4716520; doi:10.1002/ece3.1843)
Supplement: Supplementary file 2 — Data S2. Detailed methods for molecular studies. [file ECE3-6-233-s002.pdf]

Detailed methods for molecular studies

To detect colony social form (single queen or monogyne; multiple queens or polygyne) we extracted DNA from pools of 10 workers (whole bodies) per colony and from thoraces of individual queens with the DNeasy Blood & Tissue Kit (Qiagen, Valencia, CA). We then used a *Gp-9* allele-specific PCR assay to determine social form following the protocol as described in Valles and Porter (2003). We labelled monogyne the colonies that contained only $Gp\text{-}9^{BB}$ workers, while we labelled polygyne the colonies where we detected the $Gp\text{-}9^{Bb}$ variant (Ross and Keller, 1998).

For multiplex PCR assays to detect viral infections and identify the virus types, we isolated RNA from pools of 20 workers (whole bodies) per colony and gasters from individual queens with the RNeasy Mini Kit (Qiagen, Valencia, CA). Thereafter we performed reverse-transcription (RT) PCR reactions following the protocol developed by (Valles et al., 2009) with minor modifications. We started from 0.5 µl of total RNA to obtain cDNA with the SuperScript® III First-Strand Synthesis SuperMix (Invitrogen-Life Technologies, Carlsbad, CA, USA) and using Oligo(dt) primers. Multiplex PCR reactions were run with primers specific for each virus and *Gp-9* primers as an internal control. Electrophoresis of resulting PCR products on a 1% agarose gel allowed the separation and identification of size-specific bands for each virus (Valles et al., 2009).

Viral copies were quantified using quantitative real-time PCR (QPCR) and total RNA isolated as described above (but from whole bodies). We performed an additional step with RNase-Free DNase (Qiagen) to avoid contamination of RNA samples with genomic DNA. cDNA was synthesized as above but starting from 100 ng of RNA and using Random Hexamers instead of Oligo(dt) primers. The cDNA then was diluted 2(x) with ultra-pure water. QPCR reactions were run with specific primers for the SINV-1 and SINV-2 viruses (Hashimoto et al., 2007, Hashimoto and Valles, 2008) on an ABI Prism 7900 sequence detector set for absolute quantification. Amplifications were performed in a 10µl reaction mixtures containing 5µl of 2x SYBR Green Master Mix (Applied Biosystems-Life Technologies, Carlsbad, CA, USA), 1µl of each primer (10µM) and 2µl of cDNA at the following conditions: 50°C for 2 min, 95°C for 2 min, followed by 40 cycles of 95°C for 15 sec, 64°C for 15 sec and 72°C for 1 min. Amplification fidelity was confirmed after QPCR by determining the melting temperature with the dissociation step of 95°C for 15 sec and 60°C for 15 sec. Standard curves for both viruses were constructed from plasmid clones of the corresponding genomic regions using a copy number range of 30 to 300,000 copies: plasmid clones were kindly provided by Dr. Steve Valles (USDA-ARS Gainesville, Florida). Reaction efficiency was determined by regressing C_T values against the template copy number (log) and calculated according to the formula [$E = (10^{-1/slope}) - 1$] (Klein et al., 1999). Reaction efficiencies routinely exceeded 93%.

For microarray analyses we used a platform that includes 51,531 probes matching unique transcripts obtained from the sequencing of the *S. invicta* genome (Wurm et al., 2011) plus additional sequences from transcriptomic studies. Hierarchical Clustering (Ward method) of significantly differentially regulated genes was performed in JMP Pro 10.0 (SAS, Cary, NC). We used GENESIS 1.7.6 (Graz, Austria) to cluster differentially regulated genes based on average linkage and to perform k-means clustering (Sturn et al., 2002). Pairwise comparisons between treatment groups were performed using Venny (http://bioinfogp.cnb.csic.es/tools/venny/index.html). To perform Gene Ontology (GO) analyses, we obtained *Drosophila melanogaster* ortologs with BLAST (http://blast.ncbi.nlm.nih.gov/Blast.cgi) for fire ant genes that were significantly differentially expressed between treatments and we operated functional annotation clustering in DAVID version 6 (Huang et al., 2009a, Huang et al., 2009b) with medium stringency and a cutoff of $P < 0.05$. Overrepresented biological functions (enrichment analysis) were identified by comparing the annotation composition in our list of differentially expressed genes to that of a

population background composed by all the fire ant genes with *Drosophila* orthologs that were included in the statistical analysis.

Finally, for comparative analyses with honey bees infected by Israeli Acute Paralysis Virus (2015) we obtained lists of *Drosophila* orthologs for significantly differentially expressed genes from both studies and we overlapped these lists using Venny (Oliveros, 2009). Thereafter we used a Hypergeometric test to assess whether the number of overlapping genes was higher than expected by chance (http://nemates.org/MA/progs/overlap_stats.html).

References

GALBRAITH, D. A., YANG, X., NIÑO, E. L., YI, S. & GROZINGER, C. 2015. Parallel Epigenomic and Transcriptomic Responses to Viral Infection in Honey Bees (*Apis mellifera*). *PLoS Pathog,* 11, e1004713.

HASHIMOTO, Y. & VALLES, S. M. 2008. Infection characteristics of *Solenopsis invicta* virus 2 in the red imported fire ant, *Solenopsis invicta*. *Journal of invertebrate pathology,* 99, 136-140.

HASHIMOTO, Y., VALLES, S. M. & STRONG, C. A. 2007. Detection and quantitation of *Solenopsis invicta* virus in fire ants by real-time PCR. *Journal of Virological Methods,* 140, 132-139.

HUANG, D. W., SHERMAN, B. T. & LEMPICKI, R. A. 2009a. Bioinformatics enrichment tools: paths toward the comprehensive functional analysis of large gene lists. *Nucleic Acids Research,* 37, 1-13.

HUANG, D. W., SHERMAN, B. T. & LEMPICKI, R. A. 2009b. Systematic and integrative analysis of large gene lists using DAVID bioinformatics resources. *Nature Protocols,* 4, 44-57.

KLEIN, D., JANDA, P., STEINBORN, R., MÜLLER, M., SALMONS, B. & GÜNZBURG, W. H. 1999. Proviral load determination of different feline immunodeficiency virus isolates using real-time polymerase chain reaction: Influence of mismatches on quantification. *ELECTROPHORESIS,* 20, 291-299.

OLIVEROS, J. 2009. VENNY. An interactive tool for comparing lists with Venn Diagrams. 2007.

ROSS, K. G. & KELLER, L. 1998. Genetic control of social organization in an ant. *Proceedings of the National Academy of Sciences,* 95, 14232-14237.

STURN, A., QUACKENBUSH, J. & TRAJANOSKI, Z. 2002. Genesis: cluster analysis of microarray data. *Bioinformatics,* 18, 207-208.

VALLES, S. M. & PORTER, S. D. 2003. Identification of polygyne and monogyne fire ant colonies (*Solenopsis invicta*) by multiplex PCR of *Gp-9* alleles. *Insectes Sociaux,* 50, 199-200.

VALLES, S. M., VARONE, L., RAMÍREZ, L. & BRIANO, J. 2009. Multiplex detection of *Solenopsis invicta* viruses-1,-2, and-3. *Journal of virological methods,* 162, 276-279.

WURM, Y., WANG, J., RIBA-GROGNUZ, O., CORONA, M., NYGAARD, S., HUNT, B. G., INGRAM, K. K., FALQUET, L., NIPITWATTANAPHON, M., GOTZEK, D., DIJKSTRA, M. B., OETTLER, J., COMTESSE, F., SHIH, C. J., WU, W. J., YANG, C. C., THOMAS, J., BEAUDOING, E., PRADERVAND, S., FLEGEL, V., COOK, E. D., FABBRETTI, R., STOCKINGER, H., LONG, L., FARMERIE, W. G., OAKEY, J., BOOMSMA, J. J., PAMILO, P., YI, S. V., HEINZE, J., GOODISMAN, M. A. D., FARINELLI, L., HARSHMAN, K., HULO, N., CERUTTI, L., XENARIOS, I., SHOEMAKER, D. & KELLER, L. 2011. The genome of the fire ant *Solenopsis invicta*. *Proceedings of the National Academy of Sciences of the United States of America,* 108, 5679-5684.
