# Supplementary material for: Dynamic changes in host–virus interactions associated with colony founding and social environment in fire ant queens (Solenopsis invicta)
Source: Ecol Evol. 2015 Dec 29;6(1):233–44. doi: 10.1002/ece3.1843 (PMC4716520; doi:10.1002/ece3.1843)
Supplement: Supplementary file 3 — Figure S1. Survival curves for founding queens during the first month of activity. [file ECE3-6-233-s003.pdf]

Dynamic changes in host-virus interactions associated with colony founding and social environment in fire ant queens (*Solenopsis invicta*)

Fabio Manfredini, DeWayne Shoemaker, Christina M. Grozinger; Ecology and Evolution

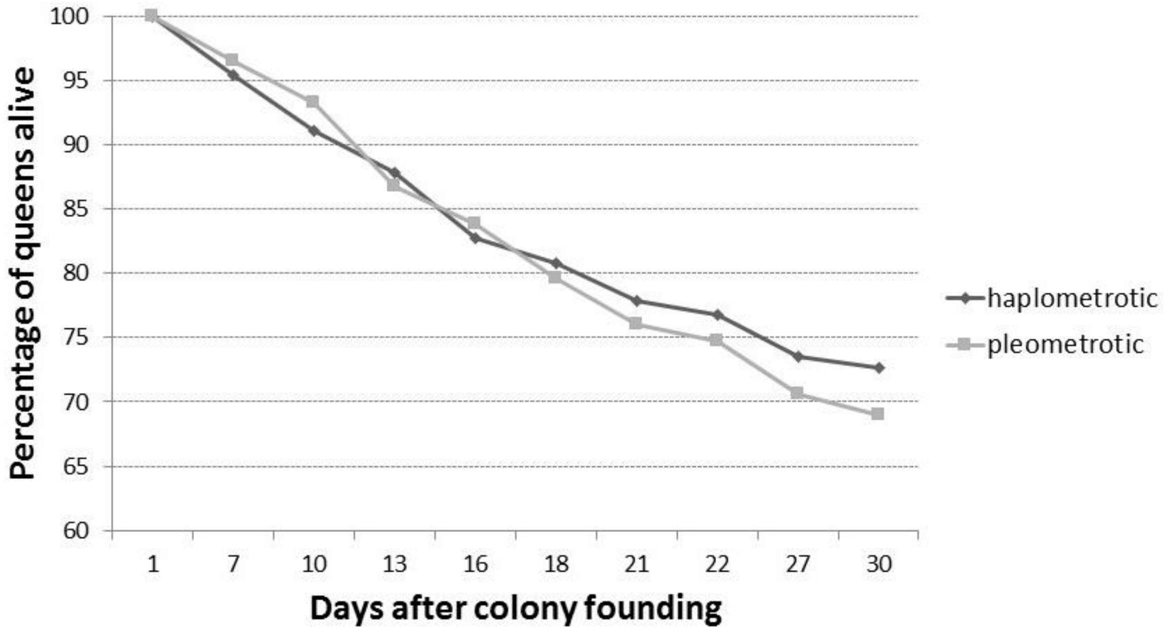

Figure S1. Survival curves for founding queens during the first month of activity. The chart shows separate analyses for single and paired queens (haplometrotic and pleometrotic, respectively).
