# Supplementary material for: Dynamic changes in host–virus interactions associated with colony founding and social environment in fire ant queens (Solenopsis invicta)
Source: Ecol Evol. 2015 Dec 29;6(1):233–44. doi: 10.1002/ece3.1843 (PMC4716520; doi:10.1002/ece3.1843)
Supplement: Supplementary file 4 — Figure S2. Copy numbers of SINV‐1 and SINV‐2 in queens that harbour one of the viruses or both of them as detected by quantitative real‐time PCR. [file ECE3-6-233-s004.pdf]

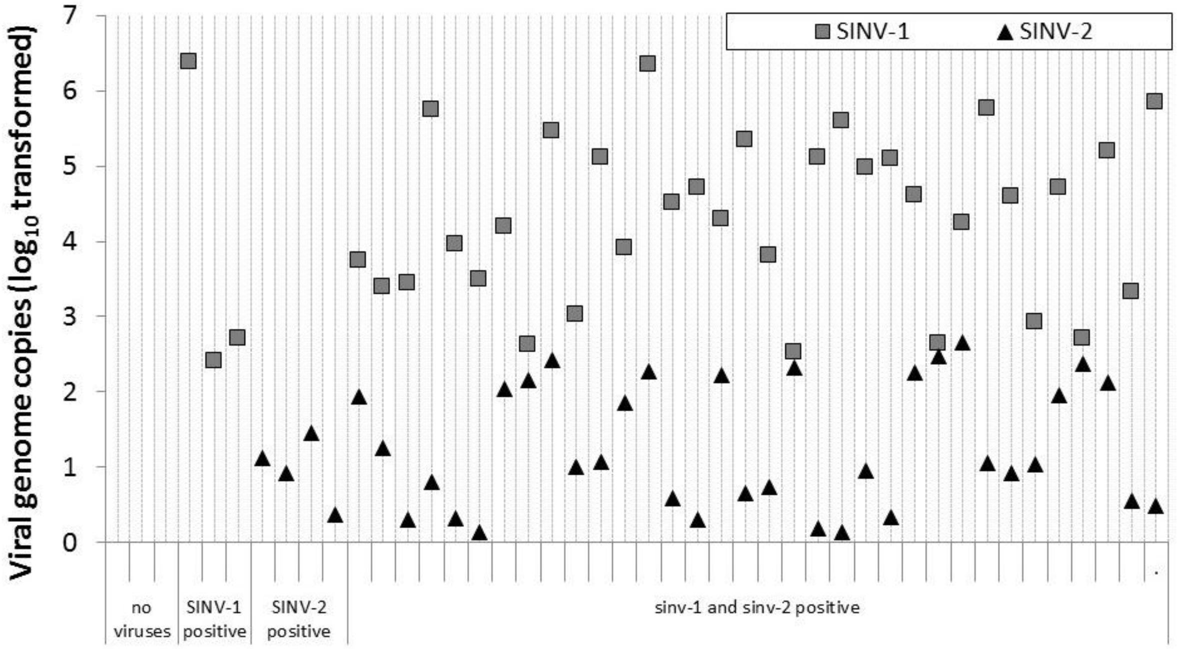

Figure S2. Copy numbers of SINV-1 and SINV-2 in queens that harbour one of the viruses or both of them as detected by quantitative real-time PCR. We used total RNA isolated from individual queens for this analysis.

Dynamic changes in host-virus interactions associated with colony founding and social environment in fire ant queens (*Solenopsis invicta*)

Fabio Manfredini, DeWayne Shoemaker, Christina M. Grozinger; Ecology and Evolution

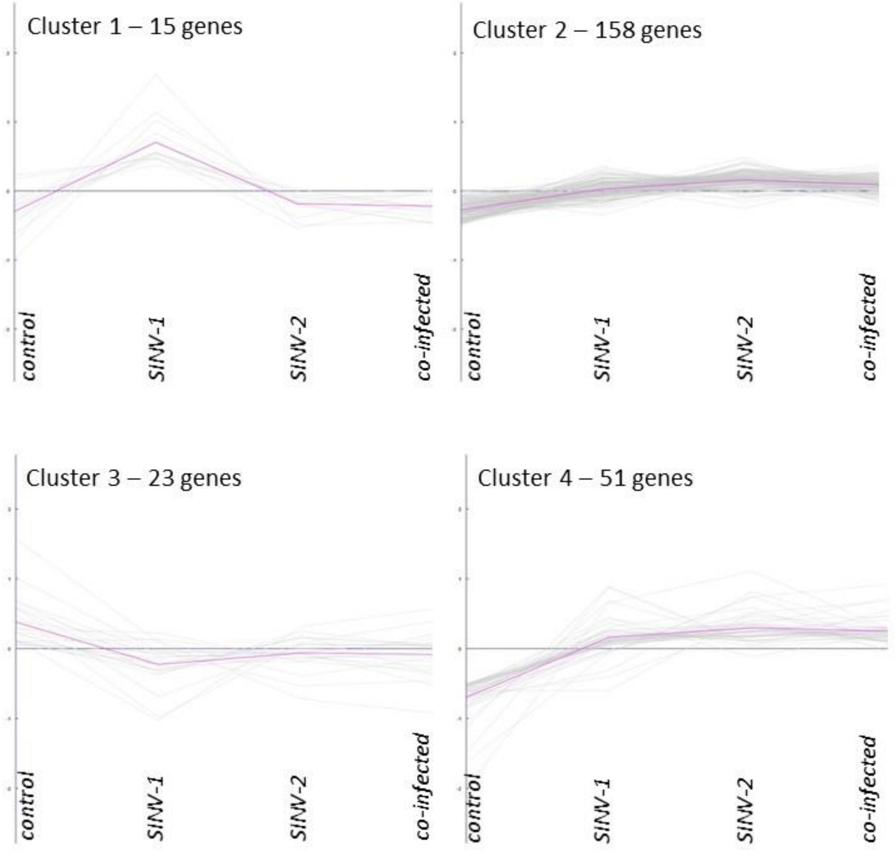

Figure S3. K-means clustering analysis of significantly differentially expressed transcripts. K-means clustering analysis was performed on the 247 transcripts that were significantly differentially expressed (*FDR* < 0.05) in at least one of the pairwise comparisons across treatment groups. Genes were grouped in four clusters according to their pattern of expression across the four treatment group control, SINV-1, SINV-2 and co-infected. **Cluster 1** (15): transcripts that were up-regulated in SINV-1 and down-regulated in all other groups; **cluster 2** (158): transcripts that were up-regulated in SINV-2; **cluster 3** (23): transcripts that were up-regulated in control and down-regulated everywhere else; **cluster 4** (51): transcripts that were up-regulated in all infected queens.
