# Supplementary material for: Dynamic changes in host–virus interactions associated with colony founding and social environment in fire ant queens (Solenopsis invicta)
Source: Ecol Evol. 2015 Dec 29;6(1):233–44. doi: 10.1002/ece3.1843 (PMC4716520; doi:10.1002/ece3.1843)
Supplement: Supplementary file 5 — Figure S3. K‐means clustering analysis of significantly differentially expressed transcripts. [file ECE3-6-233-s005.pdf]

Dynamic changes in host-virus interactions associated with colony founding and social environment in fire ant queens (*Solenopsis invicta*)

Fabio Manfredini, DeWayne Shoemaker, Christina M. Grozinger; Ecology and Evolution

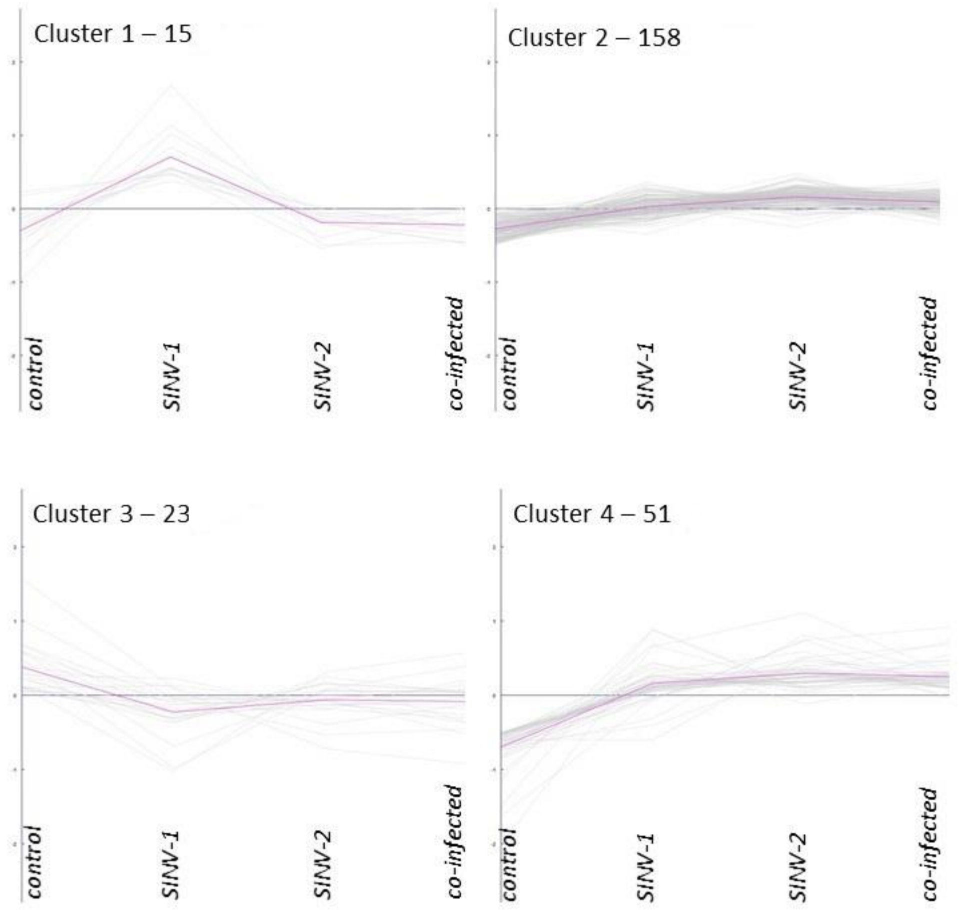

Figure S3. K-means clustering analysis of significantly differentially expressed transcripts. K-means clustering analysis was performed on the 247 transcripts that were significantly differentially expressed (*FDR<0.05*) in at least one of the pairwise comparisons across treatment groups. Genes were grouped in four clusters according to their pattern of expression across the four treatments, i.e. control, SINV-1, SINV-2 and co-infected. **Cluster 1**: 15 transcripts that were up-regulated in SINV-1 and down-regulated in all other groups; **cluster 2**: 158 transcripts that were up-regulated in SINV-2; **cluster 3**: 23 transcripts that were up-regulated in control and down-regulated everywhere else; **cluster 4**: 51 transcripts that were up-regulated in all infected queens.
